# Stable isotopes reveal seasonal dietary responses to agroforestry in a venomous mammal, the Hispaniolan solenodon (*Solenodon paradoxus*)

**DOI:** 10.1002/ece3.8761

**Published:** 2022-03-24

**Authors:** Alexis M. Mychajliw, Juan N. Almonte, Pedro A. Martinez, Elizabeth A. Hadly

**Affiliations:** ^1^ 6429 Departments of Biology and Environmental Studies Middlebury College Middlebury Vermont USA; ^2^ 6429 Department of Biology Stanford University Stanford California USA; ^3^ Museo Nacional de Historia Natural “Prof. Eugenio de Jesús Marcano” Santo Domingo Dominican Republic; ^4^ Escuela de Biología Universidad Autónoma de Santo Domingo Santo Domingo Dominican Republic; ^5^ 6429 Woods Institute of the Environment Stanford University Stanford California USA

**Keywords:** agroforestry, Caribbean, insectivory, museum collections, small mammal, stable isotope analysis

## Abstract

While trends in tropical deforestation are alarming, conservation biologists are increasingly recognizing the potential for species survival in human‐modified landscapes. Identifying the factors underlying such persistence, however, requires basic ecological knowledge of a species’ resource use. Here, we generate such data to guide conservation of an understudied venomous mammal, the Hispaniolan solenodon (*Solenodon paradoxus*), that occupies a mosaic landscape of agriculture and forest fragments in the western Dominican Republic. Using feces collected in both wet and dry seasons, we found significant differences in the stable isotope values of carbon (δ^13^C) between pasture (−24.63 ± 2.31‰, Las Mercedes) and agroforestry (−28.07 ± 2.10‰, Mencia). Solenodon populations in agricultural areas occupied wider isotopic niche spaces, which may be explained by more diverse resource within these patches or individuals combining resources across habitats. We detected elevated δ^15^N values in the dry season of pasture areas (8.22 ± 2.30‰) as compared to the wet season (5.26 ± 2.44‰) and overall narrower isotopic niche widths in the dry season, suggestive of the impacts of aridity on foraging behavior. Our work highlights the importance of considering a more nuanced view of variations in ‘modified’ or “agricultural” landscapes as compared with strictly protected national parks. We suggest that seasonal differences in foraging should be considered as they intersect with landscape modification by landowners for maintaining resources for focal consumers. This work adds to a growing body of literature highlighting that fecal stable isotopes are a non‐invasive and cost‐effective monitoring tool that is particularly well‐suited for cryptic small mammal species, ensuring actionable and evidenced‐based conservation practices in the tropic's rapidly changing landscapes.

## Graphical abstract

The Hispaniolan Solenodon (*Solenodon paradoxus*) is a venomous mammal endemic to the Dominican Republic and Haiti. Stable isotope analysis of fecal samples, with a historical hair reference, show how solenodons may alter their resource use in response to agroforestry and associated land cover changes across seasons.

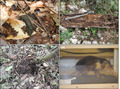

## 1 INTRODUCTION

The tropical landscapes of the future will look different from those of the recent past, consisting of vegetation mosaics that reflect varying levels of anthropogenic activity and economic pressures (Mendenhall et al., 2014; Pendrill et al., 2019; Tilman et al., 2017). A growing body of research suggests that certain species may survive and even thrive in these human‐dominated landscapes based on their traits and evolutionary history (Frank et al., 2017; Hirschfeld & Rödel, 2017). Many of these attributes—trophic position, dietary flexibility, feeding guild, niche breadth—are intimately linked to how species use resources (Solari et al., 2016). Assessing whether a species’ resource base is impacted by land use change, and if so, whether the species can utilize these new resources, can provide critical planning insight for supporting species and populations that occur outside of core protected areas in the tropics (Frishkoff et al., 2019).

While in general small mammals are expected to be less vulnerable to extinction than larger mammals (Cardillo et al., 2008), many species have restricted geographic ranges that can be easily wiped out by landscape alterations (e.g., Cervantes & Guevara, 2010) and island endemics have unique evolutionary histories with additional vulnerabilities, such as reduced reproductive rates (Lyons et al., 2016). The present‐day “security” of small mammal conservation risk contrasts sharply with the massive Holocene extinctions of insular small mammals—many associated with colonial agricultural practices (Cooke et al., 2017). For example, the Caribbean was once a hotspot of small mammal evolutionary diversity, but of the ~130 described species just 13 terrestrial mammals and 60 bats persist today following multiple waves of anthropogenic extinction both before and after European arrival (Turvey & Fritz, 2011; Turvey et al., 2017). Here, we evaluate how landscape change on the Caribbean island of Hispaniola (Dominican Republic and Haiti) is impacting one of the island's two remaining endemic terrestrial mammals, the Hispaniolan Solenodon (Eulipotyphla: *Solenodon paradoxus*).

The solenodon is a nocturnal species so elusive that it was once considered easier to find a “ghost” (Verrill, 1907), and the lack of natural history data hinders assessment of their conservation needs (Rupp & Leon, 2009). If phylogenetically conserved, the diet of solenodons should resemble its relatives such as hedgehogs, which rely heavily on abundant invertebrate prey, supplemented occasionally by vertebrate prey and plants (Jones et al., 2005). On the other hand, the legacy of millions of years of insular evolution has resulted in a departure of life history traits from the typical small‐bodied mammalian insectivore that breeds rapidly, has large litter sizes, and has a short lifespan (Symonds, 1999): the solenodon attains a relatively large body size of ~1 kg, births only two‐yearly litters of one to two young with a prolonged lactation period of 2–3 months, and is social, with family groups of up to six individuals sharing a burrow (Casewell et al., 2019; Eisenberg, 1975; Ottenwalder, 1991). Thus, the solenodon potentially has characteristics of both vulnerable species (slow reproductive rate, restricted distribution, evolution in absence of mammalian carnivores) and resilient species (a generalist, omnivorous diet, and nocturnal habits) (Liow et al., 2009). Virtually all known descriptions of the species’ feeding ecology are derived from captive conditions in which they have typically been fed a diet heavy in vertebrate‐derived proteins such as horse meat, beef, 2‐ to 3‐day‐old chicks, skinned mice, eggs, and milk (Ottenwalder, 1991, 1999); Allen (1910) notes that “they eat greedily of chopped meat.” However, the majority of these items would not naturally be encountered, and the small number of wild solenodon observations instead emphasize a diet of invertebrates, such as Verrill’s (1907) early description of the animal as “rooting in the earth and cultivated grounds, tearing rotten logs and trees to pieces with its powerful front claws, and feeding on ants, grubs, insects, vegetables, reptiles, and fruit… on several occasions it has been known to enter the houses in search of roaches and other vermin.” Accounts of the solenodon's extant sister lineage, the Cuban almiqui (*Atopogale cubana*), similarly emphasize a reliance on insects and worms in the wild (Echenique‐Díaz et al., 2014).

Recent habitat surveys have observed solenodons in forest fragments both within and outside of protected areas throughout the island of Hispaniola (Turvey et al., 2017). Sixty percent of the Dominican Republic's original forest was cut between 1930 and 1980 CE and replaced by oil palm, sugarcane, cacao, pastures, coffee, and human settlements (Bolay, 1997). Recent political instability and natural disasters have led to significant increases in human movement from Haiti to the Dominican Republic, resulting in uneven deforestation and disturbance both near and within national parks as a result of illegal charcoal harvest, fire, and small‐scale subsistence agriculture (Lloyd & León, 2019; Pasachnik et al., 2016). These hotspots of rapid and unregulated deforestation overlap with one of the last remaining refuges for the solenodon, the La Selle‐Bahoruco‐Jaragua‐Enriquillo UNESCO Biosphere Reserve (Kennerley et al., 2019).

Stable isotopes provide an important toolkit to elucidate how species diets and interactions respond to deforestation and disturbance. Carbon stable isotopes (δ^13^C) reveal differences in how species rely on C3 plants (Calvin–Benson cycle, δ^13^C value range −35 to −20‰) or CAM/C4 plants (Crassulacean acid metabolism/Hatch‐Slack pathway, δ^13^C value range −15 to −7‰), and thus whether they move between open and closed habitats (Ben‐David & Flaherty, 2012; DeNiro & Epstein, 1978; Giroux et al., 2015). Nitrogen is enriched with each trophic transfer upwards to consumers (Vanderklift & Ponsard, 2003) and thus stable nitrogen isotopes (δ^15^N) can illustrate the trophic position of an organism within its food chain, but also can reflect its level of nutritional stress (McCue & Pollock, 2008) and aridity conditions (Styring et al., 2016). In forested environments (e.g., preserves and fragments) we would expect to identify consumption of C3 plants (more negative δ^13^C values) and an intact, complex trophic structure that has stepwise increases in δ^15^N from herbivores to top predators following typical bioaccumulation processes. In areas that are modified and/or have agricultural activities, studies have detected incorporation of C4 foods as either crops or agricultural weeds (more positive δ^13^C values) and a modified trophic structure in which δ^15^N values do not increase in a stepwise fashion, but instead fluctuate depending on the anthropogenic food that consumers opportunistically eat and whether it has been supplemented by fertilizers (White et al., 2012). Combining foods from multiple vegetation and potential fertilizer baselines in human‐modified landscapes can result in consumer populations that have more individually variable diets, and as a result, occupy a wider isotopic niche space than populations of the same species occupying intact landscapes (Magioli et al., 2019).

Different tissues provide different windows of insight: hair provides an integrated signal of the resources used to build keratin across the hair follicle's growth, generally 30–60 days (Ben‐David & Flaherty, 2012), whereas feces reflect changes in diet on a day‐to‐day and even hourly basis depending on an animal's metabolic rate (Montanari, 2017; Salvarina et al., 2013; Sponheimer et al., 2003; Tieszen et al., 1983). Tissues such as bone, on the other hand, can provide a window into the nutrition of an organism across much of its lifespan, a timescale which may not provide resolution into differing responses to habitat disturbance and intra‐seasonal variation (Ben‐David & Flaherty, 2012). As feces contain undigested portions of food items (Sponheimer et al., 2003), fecal isotopes are useful for documenting feeding behavior rather than nutritional intake and tissue synthesis (Blumenthal et al., 2012; Phillips & O'Connell, 2016; Salvarina et al., 2013). The non‐invasive nature of fecal sampling is well suited to working with elusive species, and stable isotopes of feces have been applied in studies of wild primates (Blumenthal et al., 2012; Codron et al., 2008; Phillips & O'Connell, 2016), otters (Franco et al., 2013), whales (Arregui et al., 2018), South American felids (Crowley et al., 2019; Magioli et al., 2014), coyotes (Reid & Koch, 2017), African savannah herbivores (Codron & Codron, 2009; Codron et al., 2011), and bats (Lam et al., 2013; Moyo & Jacobs, 2020; Painter et al., 2009; Royer et al., 2015), among others.

**FIGURE 1:**
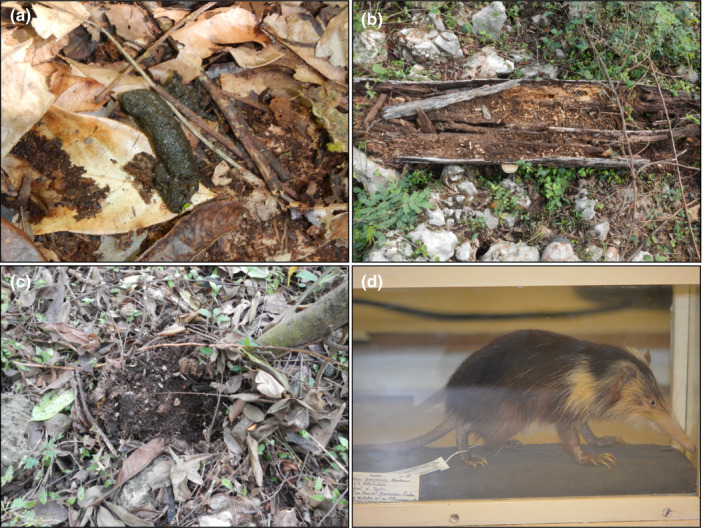
(a) An extremely fresh solenodon fecal sample. Typical signs of solenodon foraging include digging in leaf litter (b) and rotten logs (c) with their probing, ball‐and‐socket jointed nose and robust and clawed forelimbs. (d) A Cuban almiqui (*Atopogale cubanus*) at the Smithsonian National Museum of Natural History—this species is extremely rare in museum collections

We used fecal stable isotopes (δ^13^C and δ^15^N) to characterize solenodon foraging behavior across seasons and land use types in a forest‐agricultural mosaic landscape (Figure 1a). We assessed dietary variation both across seasons and across forest, pasture, and cropland sites, and explored whether agricultural populations exhibited greater isotopic niche widths as a reflection of increased resource heterogeneity with access to different invertebrate prey. We opportunistically processed historical solenodon hairs as a reference set of the natural range of variation for a more typically used tissue and to provide a foundation for future isotopic studies as a comparative baseline of anthropogenic conditions. Together, these data represent a window into solenodon diet and behavior of immediate application to both in and ex situ conservation efforts, as well as a monitoring strategy that can be applied to other elusive species globally.

## 2 MATERIALS AND METHODS

### 2.1 Sampling area and local vegetation

**FIGURE 2:**
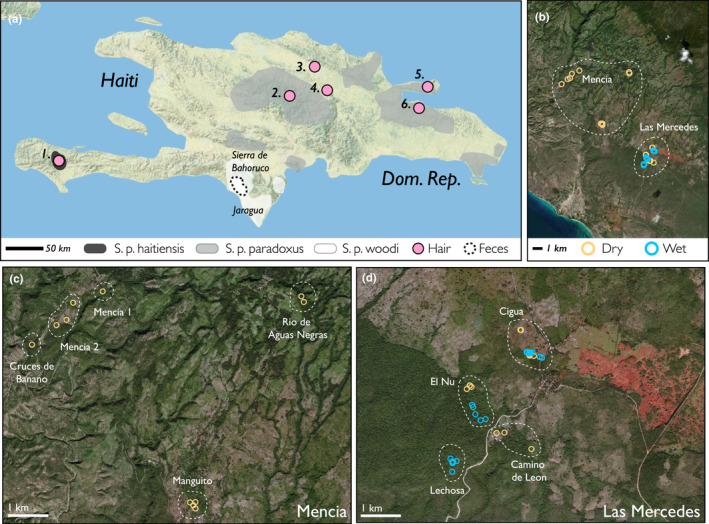
(a) The Caribbean island of Hispaniola. Extant subspecies ranges of *Solenodon paradoxus* and color‐coded by subspecies: dark gray is *S*. *paradoxus haitiensis*, light gray is *S*. *paradoxus paradoxus*, and white is *S*. *paradoxus woodi* (redrawn from Turvey et al., 2017). Approximate hair sample locations are shown in purple (Haiti: 1. Sud; Dominican Republic: 2. Cordillera Central, 3. Santiago de los Caballeros, 4. La Vega, 5. Samana, 6. Sabana del Mar). The region of fecal sample collection is encircled by a dashed line. (b) The area of fecal sample collection showing Mencia and Las Mercedes; orange = dry season collection, blue = wet season collection. Close up of (c) Mencia and (d) Las Mercedes, with dashed lines indicating different landowners and local place names. Maps produced using ESRI ArcGIS, map credits: CGIAR, Earthstar Geographics, Garmin, USGS, METI/NASA, HERE, FAO

Solenodon fecal samples were collected from Mencia and Las Mercedes in Pedernales Province, Dominican Republic (~18°08′N, 71°39′W; Figure 2), along the southwestern edge of Parque Nacional Sierra de Bahoruco (PNSB) at ~300–400 m asl (Kennerley et al., 2019; Figure 2a). PNSB is approximately 1000 km^2^ in area and, through the transboundary La Selle‐Bahoruco‐Jaragua‐Enriquillo UNECSO Biosphere Reserve, connects to ecologically important mountain ranges in Haiti (Figure 2b–d). Geologically, the region is dominated by limestone karst of Miocene age, with naturally forming Swiss‐cheese‐like caverns and holes that form important ‘burrow’ habitat for native species including solenodons and iguanas (Ottenwalder, 1999). Villages in the area rely on subsistence agriculture, animal husbandry, and small‐scale cash crops as well as the direct harvest of forest resources (Lloyd & León, 2019).

Our focal region consists of a mixture of unmanaged pastures, shade coffee, small‐scale mixed cropland and primary and secondary forest fragments. The native vegetation of this region is C3‐dominated mid‐elevation broadleaf forest with extensive leaf litter. Endemic plants clustered on exposed limestone and variable canopy height with epiphytes and lianas (Fisher‐Meerow & Judd, 1989; León et al., 2011; Ottenwalder, 1999). Before 21st century deforestation, the most abundant vegetation families at Las Mercedes were Malvaceae, Euphorbeaceae, and Fabaceae (Fisher‐Meerow & Judd, 1989), with the leguminous species *Acadia macracantha* and *Prosopis juliflora* common in disturbed areas and *Capparis ferruginea* and *Zizyphus rignoni* found in undisturbed areas. The most common shrubs are *Comocladia dodonaea*, *Guaiacum sanctum*, *Abutilon abutiloides*, and *Cryptorhiza haitiensis*. Ottenwalder (1999) documented a large number of trees with a diameter at breast height >15cm including *Vitex divaricata*, *Bursera simaruba*, *Ficus citrifolia*, and *Rauvolfia nitida*. Thus, solenodons consuming resources from native forest vegetation, dominated by C3 trees, shrubs, and grasses (typical δ^13^C value range −35 to −20‰, though given local climatic conditions (Table S1) values upwards of −25‰ are uncommon) should exhibit more negative δ^13^C values. Cultivated C3 plants in the study area include squash, cacao, coffee, and fruits such as mangoes and plantains, and given fertilizer use, would be enriched relative to wild C3 vegetation. C4 plants in the Dominican Republic are rare but can be found in littoral areas or at low elevation open environments (e.g., plants within the families Poacecae and Cyperaceae), but overall C4 contributions to native vegetation are considered minimal outside of anthropogenic contexts such as maize (introduced by Indigenous people), amaranths, and weeds accompanying agricultural activities and disturbances (Lane et al., 2009).

Our work builds on several prior surveys that confirmed the presence of solenodons (Kennerley et al., 2019; Pozo‐Rodríguez, 2011; Turvey et al., 2014; León et al., 2011, 2014). While both Mencia (Figure 2c) and Las Mercedes (Figure 2d) consist of mosaics of natural vegetation, secondary growth, and agriculture (Kennerley et al., 2019), at the time of our sampling, agricultural localities in Las Mercedes had less forest cover and high livestock pasture activity, whereas those in Mencia were more typical of shade grown crops. Neither locality had cultivated CAM plants such as pineapple or prickly pear cactus. Though we group localities within our focal region into “agriculture” and “forest” for ease of contrast, these are relative terms, as the entire area is considered disturbed and used by humans outside of the strict core protected area of the UNESCO Biosphere Reserve; therefore, our analyses compare degrees of disturbance within an overall altered landscape.

### 2.2 Feces collection and processing

Solenodons can be found living in groups within limestone karst burrows and holes within dead trees, with multiple related individuals using the same landscape in an average home range of ~156,700 m^2^ (Kennerley, 2014; Kennerley et al., 2019). Solenodon feces are easy to distinguish, as they are the only mammalian insectivore on the island and their feces has a visibly high abundance of chitinous material and a goat‐like odor (Figure 1a). Aligning with prior radio‐collar studies (Kennerley et al., 2019), samples were collected in 2015 from the dry season from both Las Mercedes and Mencia, and the wet season from Las Mercedes (Figure 2b; see Table S1 for average environmental conditions).

We identified active solenodon foraging sites using “nose‐pokes,” which are conical holes in the soil and leaf litter (Figure 1b), as well as other foraging modes such as rooting through rotting wood (Figure 1c). The social behavior of solenodons in foraging as a group, sharing a burrow, and defecating together suggests that we captured feces from multiple individuals (Kennerley, 2014; Ottenwalder, 1991). Our fecal sampling approach was designed to minimize the potential for pseudoreplication by collecting fresh feces from a single area within the same day, rather than returning to the same burrow over multiple days, such that a single individual would not be producing the same fecal samples over time. Each fecal sample was categorized as being from an “agriculture” or “forest” context upon collection based on local conditions—for example, the nearby presence of livestock, crops, or recent preparation of soil for planting through burning or tilling.

Fifty fecal samples were stored with silica beads and the outside layer was removed during processing to avoid surface contaminants. Feces were then ground to a fine powder and homogenized using a mortar and pestle. We analyzed fecal isotopes at the population level as grouped by landscape category and season, rather than focally following individuals, consistent with prior isotopic monitoring of wild mammals (Blumenthal et al., 2012; Phillips & O'Connell, 2016).

### 2.3 Historical hair samples

We sampled guard hairs from 32 individuals from several museum collections (American Museum of Natural History, Museum of Comparative Zoology, Florida Museum of Natural History, the Smithsonian National Museum of Natural History) spanning 1890–1975 CE (Figure 2d). The majority of samples come from La Vega, Dominican Republic from 1908 and 1916, with other isolated samples from areas in eastern Hispaniola, one from Haiti, and three captive individuals from the New York Zoological Society (now Bronx Zoo) in the 1960s. For comparative purposes, we also analyzed two samples of the Cuban almiqui (Sato et al., 2016; Figure 1d). We washed ~1 mg of hair in a 2:1 chloroform/methanol solution to remove lipids and contaminants, followed by methanol and water (Oelze, 2016). After decanting, hairs were dried under a fume hood for 48 h.

### 2.4 Stable isotope analysis

Samples were packed into 3.5 × 5 mm tin capsules and flash combusted on a Thermo Finnigan Deltaplus XL interfaced with a Costech Environmental Analyzer at Stanford University's Stable Isotope Biogeochemistry Lab. We used multiple standards within each run, including both in‐house laboratory standards of acetanilide and bovine gelatin powder, and the international standard USGS 40 (l‐glutamic acid). We determined an external precision of <0.1‰ for δ^15^N and <0.15‰ for δ^13^C and internal precision of <0.3‰ for δ^15^N and δ^13^C. We included at least four blanks per run to detect contamination.

All results are reported using the δ notation with values in parts per mil (‰) relative to an international carbon (VPDB) and nitrogen (air) standard, where: δX (‰) = [(*R*_sample_/*R*_standard_) – 1] × 1000. Anthropogenic fossil fuel use has depleted the δ^13^C values of atmospheric CO_2_ relative to pre‐Industrial levels; therefore, we corrected all samples to 2015 with a year‐specific depletion value (Long et al., 2005) to account for the Suess effect when comparing samples collected at different times. Raw stable isotope values are reported in Table S2.

### 2.5 Statistical analyses

We used standard tests of normality, including Shapiro–Wilk tests to assess normality and *F* tests to assess variances for our δ^13^C and δ^15^N datasets, and inspected *Q*–*Q* plots. Where possible, we used parametric tests for our comparisons between individual isotopes and used a Wilcoxon rank sum test for nonparametric cases (*p* < .05 threshold). Comparing multiple groups of agricultural and forest areas across Las Mercedes and Mencia or by seasons for single isotopes, we employed a Kruskal–Wallis rank sum test of multiple comparisons followed by pairwise Wilcoxon rank sum tests to identify significant levels with a post‐hoc Benjamini & Hochberg *p* value adjustment.

We calculated niche width for different groups within sites (dry vs. wet season, forest vs. agriculture) using two metrics: the total convex hull area (TA) and the standard ellipse area (SEA) with the R package Stable Isotope Bayesian Ellipses in R, ‘SIBER’ (Jackson et al., 2011). TA measures niche width by drawing a convex hull containing all points within a group plotted in a δ^13^C–δ^15^N biplot and is sensitive to differences in sample size (Layman et al., 2007). SEA (small sample size corrected, SEA_c_) is not sensitive to sample size, and calculates the standard ellipse that contains ~40% of the data (comparable to standard deviation in univariate calculations), measured in per mil squared (‰^2^) (Jackson et al., 2011). We also calculated ellipses scaled to represent a 95% confidence ellipse of the bivariate means.

We then fit Bayesian multivariate normal distributions to each group and its posterior distribution, facilitating comparison of SEA_c_ area between groups (*n* = 10,000, burnin = 1000). To test whether ellipses (and therefore isotopic niche widths) were different between groups, we calculated the probability that the posterior distribution of a group's SEA_c_ is larger or smaller than the other: we compared the posterior draws for both groups and calculated the proportion of draws that were smaller as a proxy for the probability that one group's posterior distribution is smaller than the other (based on 10,000 draws).

In addition to calculating TA and SEA_c_ for groups, we determined δ^13^C Range (CR), δ^15^N Range (NR), Mean Distance to Centroid (CD), and Nearest Neighbor Distance (NND) to assess variation (Layman et al., 2007). We used SIBER to calculate these community ecology metrics, which implements a Bayesian inference technique to incorporate the uncertainty of centroid location in our focal communities. As defined in Layman et al. (2007), CR is calculated as the difference between the highest and lowest values of δ^13^C and reflects the diversity of primary producer resources, though caution should be taken in considering fractionation effects between trophic positions. NR is calculated as the difference between the highest and lowest values of δ^15^N and reflects the trophic diversity or trophic length in a community. CD is used to evaluate spacing within the niche. NND is a measure of clustering within a community, and SDNND can be similarly informative regarding variability of individual resource use.

### 2.6 Environmental, anthropogenic, and climatic variables

Using R packages “sp,” “raster,” “maptools,” and “dismo,” we extracted values from several datasets for the specific coordinates of each fecal sample, including: percent forest cover (satellite images of tree canopy, 30 m^2^ resolution (Hansen et al., 2013)), human population density (National Statistics Office of the Dominican Republic, township/municipality level, 1 km^2^ resolution), soil (SoilGrids, 4.5 km^2^ resolution (Leenaars et al., 2017)), geology (1:25,000,000 scale; USGS (French & Schenk, 2004)), topography (elevation, slope, aspect; USGS, ESRI), and land use type (Global Land Cover 2000, 1 km^2^ resolution (Fritz et al., 2003)). We compared these layers with our on‐the‐ground categorization of presence of cultivated crops/pasture and recent Google Earth Satellite Imagery (2017). We selected bioclimatic variables (~1 km^2^ resolution) of known importance to solenodon habitat selection based on a prior species distribution modeling study in the region (Gibson et al., 2019), including mean diurnal range (Bio2), temperature seasonality (Bio4), max temperature of warmest month (Bio5), temperature annual range (Bio7), precipitation seasonality (Bio15), and precipitation of warmest quarter (Bio18) (Hijmans et al., 2005). To compare wet and dry season samples, we also extracted sample‐specific values of WorldClim's minimum temperature (*T*_min_), maximum temperature (*T*_max_), average temperature (*T*_avg_), and precipitation for the collection month. We used a MANOVA to compare these environmental, anthropogenic, and climatic variables between sampling site locations (Mencia vs. Las Mercedes) and land use categorizations (agriculture vs. forest). We incorporated temperature and precipitation values at time of sampling, rather than categorizing samples as “dry” or “wet” season. To explore factors that may influence solenodon isotopic values, we conducted stepwise regressions in R for δ^15^N and δ^13^C using the R package “MASS” using both a forward and backward search, which at each step calculates an AIC value and either includes or excludes a variable, until the best fit and most parsimonious model is found. We then used the R package “stargazer” (Hlavac, 2018) to summarize the resulting models.

## 3 RESULTS

### 3.1 Sampling site characteristics

Las Mercedes and Mencia are qualitatively similar in their soils and geologic classifications. Sites in Mencia have steeper slopes and experience a wider range in diurnal and annual temperatures and higher seasonality (MANOVA: slope (*F* = 18.90, *p* < .001), Bio2 (*F* = 18.84, *p *< .001), Bio7 (*F* = 31.66, *p *< .001), Bio4 (*F* = 11.99, *p *< .01)). Satellite‐based metrics of percent forest cover aligned well with our on‐the‐ground classifications of agriculture (pasture, cropland; <70% forest cover) (Hansen et al., 2013). Conversely, the Global Land Cover dataset was less accurate (Fritz et al., 2003), classifying all sites in Mencia as “cultivated and managed areas,” whereas sites in Las Mercedes were split between herbaceous cover (closed‐open, natural, pasture, and shrubs) and tree cover (broadleaf and evergreen); this is likely due to being out of date given rapid environmental change. Sample locations classified as agricultural or forested differed significantly in slope (*F* = 4.07, *p* < .5), aspect (*F* = 16.80, *p* < .001), forest (*F* = 1351.1, *p* < .001) (MANOVA), but not in bioclimatic variables, consistent with observations on the ground.

### 3.2 Comparing hair and fecal isotopes

Hairs ranged from δ^13^C −20.58 to −24.55‰ and δ^15^N 5.65 to 9.11‰ from localities across the Dominican Republic, with a low δ^15^N value from Haiti of 3.79‰ (Table 1). Our most useful insight into population‐level variation comes from 21 individuals collected in 1908–1916 CE from La Vega, Dominican Republic, averaging δ^13^C −22.11 ± 1.84‰ and δ^15^N 5.72 ± 0.79‰. Captive solenodons from the New York Zoological Society exhibited the most positive δ^13^C values, consistent with anthropogenic, C4‐skewed food sources (Kays & Feranec, 2011).

**TABLE 1:** Summary of stable isotope sample sizes (*n*), values of nitrogen (δ^15^N), carbon (δ^13^C), and standard deviations (SD), for historical solenodon hair samples

Region	Locality	Time	*n*	δ^13^C (‰)	δ^15^N (‰)
Dominican Republic	La Vega	1908, 1916	21	−22.11 ± 1.84	5.72 ± 0.79
	Cordillera Central	1910	1	−20.58	5.65
	Rio San Juan, Samana	1919	1	−24.55	9.11
	Santiago de los Caballeros	1936	1	−21.89	7.42
	Sabana del Mar	1937	2	−22.65 ± 0.81	7.80 ± 0.41
Haiti	Sud, Duchity	1975	1	−24.06	3.79
Captive	New York Zoological Society	1960–1970	3	−19.63 ± 1.17	7.30 ± 0.69
Cuba	Cuba	1871, 1909	2	−21.11 ± 1.60	6.19 ± 2.35

Note

All samples are *S*. *paradoxus* except those from Cuba, which are *A*. *cubana*.

Accurately comparing values across time and tissues requires multiple corrections. First, δ^13^C values were Suess‐corrected for global atmospheric ^13^C depletion using a year‐specific equation (Long et al., 2005) to the year 2015, making the range of representative Dominican Republic δ^13^C values −22.61‰ to −26.55‰ (Suess corrected), with their corresponding δ^15^N values 5.66‰ (1910, Cordillera Central) to 9.11‰ (1919, Rio San Juan Samana). Applying a hair‐diet isotopic offset for trophic discrimination (Δ_diet–hair_) from insectivorous bats (Siemers et al., 2011), the diet resources consumed by solenodons would have ranged from δ^15^N ~2.75–6.2‰ and δ^13^C −26.02 to −29.96‰, consistent with insectivory in a C3 environment. In general, feces have lower δ^13^C values and higher δ^15^N values as compared with bulk diet items (see previous reviews of wild and captive mammals (Crowley et al., 2019)). Using the average of the most appropriate available offset values for trophic discrimination (Δ_diet–feces_) – the omnivorous meerkat (Montanari, 2017), and the insectivorous greater mouse‐eared bat and greater horseshoe bat (Salvarina et al., 2013)—food items would have on average been δ^15^N 5.84‰ and δ^13^C −26.16‰, falling well within the expected range of solenodon resource values based on our historical hair assessments. Feces yielded atomic C:N ratios consistent with chitin consumption (Holden & Southon, 2016).

### 3.3 Seasonal patterns in Las Mercedes

**FIGURE 3:**
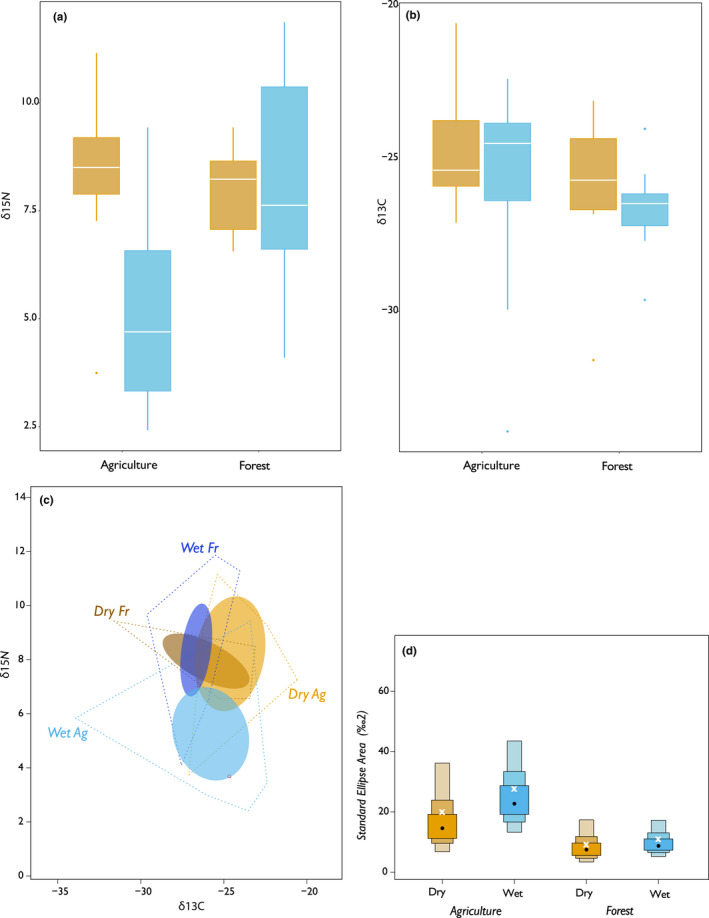
Values of (a) δ^15^N (‰) and (b) δ^13^C (‰) from Las Mercedes across wet (blues) and dry (oranges) seasons. Comparison of the (c) total area (TA) of the convex hulls and 95% confidence intervals around the bivariate means of different groupings (dry forest (dark orange), dry agriculture (light orange), wet forest (dark blue), and wet agriculture (light blue)) and (d) the Bayesian standard ellipse area (SEA_b_), with white Xs indicating the maximum likelihood estimates of SEA_c_. Sample sizes are in Table 2

We collected 14 dry season (average δ^15^N 8.09 ± 1.74‰ and δ^13^C −25.35 ± 2.59‰) and 24 wet season (average δ^15^N 6.81 ± 2.86‰ and δ^13^C −26.23 ± 2.49‰) fecal samples within the area of Las Mercedes, across the properties of several landowners (Figure 3a,b; Table 2). Both TA and SEA_c_ were consistently larger for agricultural sites as compared with forest sites across seasons (Figure 3c; Table 3). The agricultural sites across seasons also exhibited a larger δ^13^C and δ^15^N range, CD, NND, and SDNND, suggesting more variation in individual resource use (Table 3). We found that the SEA_b_ (Bayesian SEA estimate) is larger for the wet season both for agriculture (.7868 probability) and forest (.6275 probability) (Figure 3d). We found significant differences in δ^15^N values across the wet and dry seasons of agriculture and forest fecal samples in Las Mercedes (Kruskal‐Wallis, *H* = 10.40, df = 3, *p* = .0015); post‐hoc pairwise comparisons identified that δ^15^N values were significantly elevated in the dry season of agricultural sites (*p* < .05).

**TABLE 2:** Summary of stable isotope sample sizes (*n*), values of nitrogen (δ^15^N), carbon (δ^13^C), and standard deviations (SD), for solenodon fecal samples analyzed in this study

Categories		Las Mercedes			Mencia		
Season	Habitat	*n*	δ^13^C (‰)	δ^15^N (‰)	*n*	δ^13^C (‰)	δ^15^N (‰)
Dry	Agriculture	7	−24.63 ± 2.31	8.22 ± 2.30	6	−28.07 ± 2.10	5.48 ± 2.43
	Forest	7	−26.07 ± 2.82	7.95 ± 1.10	6	−26.71 ± 1.71	7.70 ± 2.51
	All	14	−25.35 ± 2.59	8.09 ± 1.74	12	−27.39 ± 1.96	6.59 ± 2.62
Wet	Agriculture	12	−25.79 ± 3.28	5.26 ± 2.44	–	–	–
	Forest	12	−26.66 ± 1.36	8.36 ± 2.43	–	–	–
	All	24	−26.23 ± 2.49	6.81 ± 2.86	–	–	–

**TABLE 3:** Metrics of isotopic niche width, as calculated in Stable Isotope Bayesian Ellipses in R SIBER, for comparing seasons in Las Mercedes and habitat types across Mencia and Las Mercedes

	Las Mercedes: dry vs. wet season				Mencia vs. Las Mercedes (dry season)			
	Agriculture		Forest		Mencia		Las Mercedes	
	Dry	Wet	Dry	Wet	Agriculture	Forest	Agriculture	Forest
TA	23.96	41.85	10.99	21.68	18.40	17.32	23.96	10.99
SEAc	19.66	27.38	8.86	10.65	18.66	16.44	19.66	8.86
CR	13.35		8.48		7.56		11.02	
NR	8.74		7.77		8.70		7.41	
CD	3.62		2.35		2.89		2.44	
NND	1.34		1.07		1.47		1.56	
SDNND	0.98		0.69		0.88		1.29	

Abbreviations: CD, mean distance to centroid; CR, carbon range; NND, nearest neighbor distance; NR, nitrogen range; SDNND, standard deviation of nearest neighbor distance; SEAc, standard ellipse area small sample size corrected; TA, total area.

### 3.4 Comparisons of Mencia and Las Mercedes in the dry season

**FIGURE 4:**
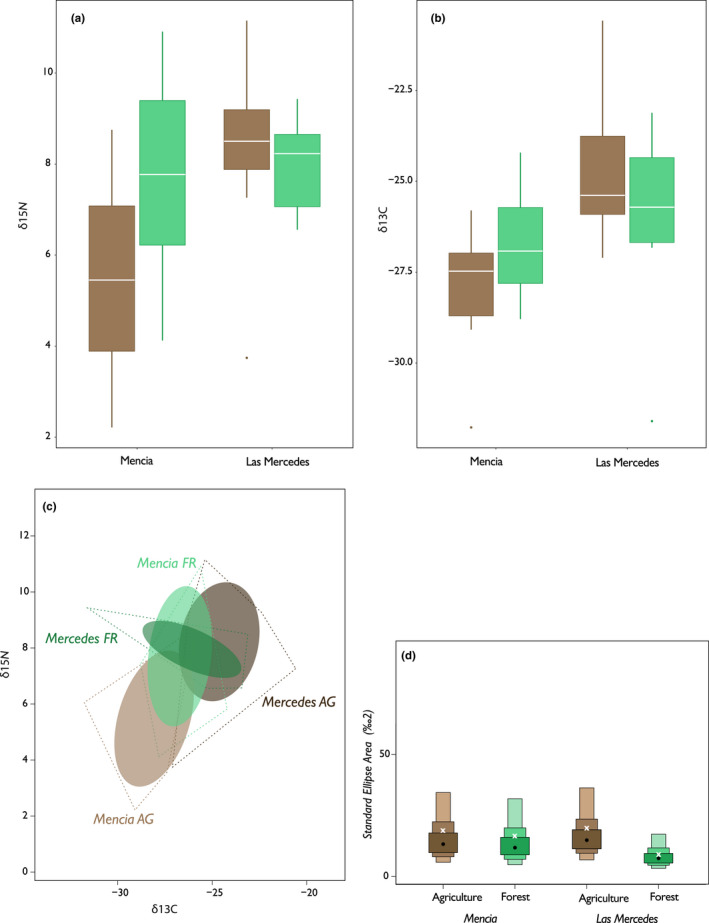
Values of (a) δ^15^N (‰) and (b) δ^13^C (‰) from Mencia and Las Mercedes across habitat types (forest = greens, agriculture = browns). Comparison of the (c) total area (TA) of the convex hulls and 95% confidence intervals around the bivariate means of different groupings (Mencia forest (light green), Mencia agriculture (light brown), Las Mercedes forest (dark green), and Las Mercedes agriculture (dark brown)) and (d) the Bayesian standard ellipse area (SEA_b_), with white Xs indicating the maximum likelihood estimates of SEA_c_. Sample sizes are in Table 2

Within the dry season, we collected 12 samples from Mencia (average δ^15^N 6.59 ± 2.62‰ and δ^13^C –27.39 ± 1.96‰) and 14 from Las Mercedes (δ^15^N 8.09 ± 1.74‰ and δ^13^C −25.35 ± 2.59‰) for Las Mercedes (Figure 4a,b; Table 2; Figure S1). We found significant differences in δ^13^C values of agriculture and forest fecal samples across Las Mercedes and Mencia (Kruskall‐Wallace, *H* = 8.08, df = 3, *p* = .04); post‐hoc pairwise comparisons identified that δ^13^C values were significantly more positive in agricultural sites in Las Mercedes as compared with Mencia (*p* < .05). TA and SEA_c_ was consistently larger for agricultural sites than forest sites in both areas (probability of Mencia forest < Mencia agriculture = 0.597, Las Mercedes forest < Las Mercedes agriculture = 0.89625) (Figure 4c; Table 3). While the δ^15^N range and CD were larger in Mencia, the δ^13^C range was larger in Las Mercedes (Table 2). Both NND and SDNND were larger for Las Mercedes, suggesting greater variation among individual resource use across the forest and agricultural areas of the site. The Mencia forest SEA_c_ was larger than that of the Last Mercedes forest (probability of Las Mercedes forest <Mencia forest = 0.81025) (Figure 4d).

### 3.5 Correlates of isotopic values

We used Pearson correlation of *r* ^2^ < .70 (Merow et al., 2013; Warren et al., 2010) to remove correlated variables and avoid collinearity (Figure S2). This left slope, aspect, Bio18, Bio2, Bio15, Bio4, mean seasonal temperature, forest cover, and human population density for use in a stepwise regression. Stepwise regressions of the best models returned by the selection procedure for δ^13^C (AIC = 90.54) and δ^15^N (AIC = 81.9) exhibited low *r* ^2^ values (.195 and .288, respectively), though identified several significant coefficients (Table S3). δ^15^N values were significantly associated with Bio2 (including mean diurnal range, *p *< .01), Bio4 (temperature seasonality, *p *< .01), mean seasonal temperature (*p *< .1), and human density (reflecting differences between Las Mercedes and Mencia, *p *< .05). δ^13^C values were significantly associated with mean seasonal temperature (*p *< .1), forest cover (*p *< .05), and human population density (*p *< .05) (Table S4).

## 4 DISCUSSION

The ability of species to persist in new ecological configurations will depend on their individualistic responses to changes in resource availability, including dietary flexibility and foraging behaviors (Castaño‐Villa et al., 2019), as well as tolerance to disturbance and human‐wildlife conflicts (Smith et al., 2018). Understanding the determinants of persistence in turn can inform monitoring strategies and help identify conservation priorities outside of protected areas and in agroforestry landscapes, where significant biodiversity conservation opportunities exist (Cassano et al., 2014; Martin et al., 2020).

Without basic natural history knowledge, we cannot adequately anticipate species responses to land use change. Based on evolutionary history, we hypothesized that the solenodon would be a generalist insectivore across varying landscapes; however, its relatively large body size (~1 kg) and potential consumption of poultry may make it more prone to targeted killings by farmers, thus changing the calculus of foraging risk for solenodons in human‐altered spaces. Historic hair samples from museum collections were consistent with published isotope ranges for insectivorous American shrew species (Baltensperger et al., 2015) and South American shrew mice and opossums (Galetti et al., 2016). In the absence of information on contemporaneous land use conditions, we are unable to meaningfully interpret the ecology of historical solenodon populations based on their hair alone, but we present these values to (1) assess general congruence with fecal isotope values and (2) provide references for future conservation work with these populations in a historical ecology lens. Our work suggests that solenodons, as generalist insectivores, can find suitable insects and other prey items (e.g., millipedes, snails, and spiders) in different vegetation contexts, suggesting that they can persist in agroforestry spaces outside of protected areas based on their ecology alone. This indication of dietary resilience suggests that loss of forest cover does not equate to a loss of resources necessary for solenodon survival. Instead, other factors, like predation by village dogs and persecution for perceived poultry depredation, appear to be the main determinant of solenodon persistence on local scales (Gibson et al., 2019; Turvey et al., 2017).

We used feces to provide isotopic snapshots of resource use and may alter their responses to disturbance and landscape modification on highly local and seasonal scales dependent on types of agricultural activity. Fecal stable isotopes have been shown to reliably distinguish between pure C3, pure C4, and mixed feeder diets (Codron et al., 2005). Our estimates of the δ^15^N and δ^13^C resources used by solenodon populations sampled via hair and feces (corrected for trophic discrimination) (Montanari, 2017; Salvarina et al., 2013; Siemers et al., 2011) broadly range from ~3 to 6‰ and ~−26 to −30‰, respectively. These values are consistent with a mostly C3 primary productivity baseline as expected for the type of anthropogenic landscape change in our region of study. While humans have been altering Hispaniolan landscapes since the mid‐Holocene (Cooke et al., 2017), the majority of stable isotope research for the region has focused on archaeological and paleontological—rather than ecological—questions. Contributions of C4 plants to animal diets, as gleaned through δ^13^C values, are regularly used to infer some type of anthropogenic influence on species such as dogs, rice rats, and hutias, given the otherwise natural rarity of C4 plants in the region and predominance of maize in human diets (e.g., Shev et al., 2021). Cooke and Crowley (2018) examined nine rodent taxa (eight of which went extinct post European arrival) from paleontological contexts on the Tiburon Peninsula in Haiti using stable isotopes of carbon and oxygen on tooth enamel; their results similarly emphasized a natural reliance on C3 plants that would also be regularly available to solenodons.

Studies of a diverse range of taxa, including Malaysian rodents (Nakagawa et al., 2007), lemurs (Crowley et al., 2013), pumas (Magioli et al., 2014), Borneo bird communities (Hamer et al., 2015), bats (Reuter et al., 2016), and even ant colonies (Woodcock et al., 2013) have reported increased δ^15^N values associated with disturbed and/or degraded forest ecosystems. We did not detect significant differences in δ^15^N values in our study, which may be because trophic interactions are still largely intact, because fertilizer is not being used, because solenodon diet may not be sensitive to disturbance, and/or because individuals are still able to utilize forest resources due to the small scale of disturbance patches in our area. Future efforts would be strengthened by simultaneously sampling vegetation and soils when conducting regular fecal monitoring to regularly track fertilizer use and fluctuations in nitrogen baselines.

Previous research in South American ecosystems has found that while more positive δ^13^C values appear common for herbivores, carnivores, and omnivores in modified landscapes, frugivores may be more likely to maintain use of C3 resources, and insectivores (Xenarthrans) appear to show no significant differences (Magioli et al., 2019). However, some Xenarthrans, particularly anteaters, are specialists on particular insect taxa and thus may not be representative of other insectivorous mammals such as solenodons. We did not detect significantly more positive δ^13^C values in agricultural sites. The forested sites of Mencia and Las Mercedes were not significantly different from each other in δ^13^C values (−26.71 ± 1.71‰ Mencia, −26.07 ± 2.82‰ Las Mercedes), reflecting the shared native vegetation of the region (Fisher‐Meerow & Judd, 1989; León et al., 2011; Ottenwalder, 1999). However, the “agriculture” category of Las Mercedes and Mencia did differ significantly from each other, with Mencia having more negative values (−28.07 ± 2.10‰) and Las Mercedes having more positive values (−24.63 ± 2.31‰). This is consistent with our on‐the‐ground assessment that while both locations would be considered agricultural or human modified landscapes in a larger regional classification, Las Mercedes’ gentler slopes are suited for pasture, and at the time of sampling had herds of livestock and a flock of turkeys. Conversely, Mencia has more topographical diversity, and at the time of sampling, was used for agroforestry purposes such as cultivating coffee and fruit trees for small‐scale consumption. Neither location had substantial monocultures of C4 crops in the immediate vicinity, though maize is grown in small family subsistence plots and agricultural weeds are present in disturbed contexts such as pastures. Thus, we stress the need for nuance when discussing disturbed or agricultural areas in tropical mosaics and broadly applying the label to any location outside of a strictly protected area, as these patches may be managed differently by different landowner preferences and local topographical conditions, facilitating different population outcomes even within “buffer” areas outside of national parks (Daily et al., 2003; Marín et al., 2009).

Our analysis of seasonal change across wet and dry seasons in Las Mercedes highlighted how landscape modification may interact with seasonal conditions to shape foraging behaviors (e.g., Teodoro et al., 2010). Feces from the dry season had significantly elevated δ^15^N values in agricultural populations as compared with the wet season (approximately 3°C temperature difference and 50 mm precipitation difference), yet there was no such difference in forest populations. Arid conditions are known to cause enriched δ^15^N values in mammals (e.g., Hixon et al., 2018; Popa‐Lisseanu et al., 2015). Further, in parallel with previous home range studies in which wet season home ranges were broader than dry season ranges (Kennerley et al., 2019), we found that the isotopic niche of the wet season was broader than the dry season. Evidence for larger wet season home ranges in solenodons is consistent with previous observations that solenodon above‐ground activity decreases during the dry season, possibly due to reduced abundance or diversity of invertebrate prey, conservation of energy, and a peak in breeding efforts (Ottenwalder, 1991).

Agricultural‐forest matrices and human‐modified landscapes can contribute to conservation goals by retaining necessary resources for consumers and/or providing them with novel resources (Brady et al., 2011; Watling et al., 2011). The present study contributes to a larger body of work suggesting that tropical agroforestry practices can support small mammal diversity (Silva et al., 2020) if species can incorporate resources from agricultural areas. We found that the population‐level isotopic niche space of solenodons was broader in agricultural sites, both in Las Mercedes and Mencia (Table 2), consistent with expectations given that consumers in human‐modified landscapes could combine foods from different vegetation and fertilizer conditions (Magioli et al., 2019). Within Las Mercedes agricultural areas, aggregated fecal samples had a larger nearest neighbor distance (NND) and standard deviation of NND (SDNND)—metrics indicative of higher individual dispersion within a population, wherein solenodons may be responding individualistically to different resources in the modified landscapes. Such a hypothesis could be addressed in the future by serially sampling tissues such as the keratin in claws, though such work would be more invasive given the need to capture and restrain individuals.

Rather than remnant forest patches forming isolated “islands” surrounded by sterile agriculture “oceans,” primary forest fragments can represent one part of a viable habitat mosaic, woven together with degraded pasture lands, regenerating vegetation, small‐scale agriculture, roads, and villages, thereby forming a continuum of spaces that species can differentially occupy based on their individual disturbance tolerances (Frishkoff & Karp, 2019). While these matrices may harbor lower species richness (Bogoni et al., 2017), they act as feeding areas for a number of species (Magioli et al., 2019). It is important to caution that while such modified areas may provide resources for solenodons and other species more broadly in the tropics, these are also spaces with greater numbers of human commensal species such as dogs, and higher levels of human‐wildlife conflict for certain taxa, especially if they are considered threats to livestock or crop production, potentially negating the benefits of resource availability (Crespin & Simonetti, 2019).

The pace of deforestation in tropical landscapes requires methodological toolkits of varying temporal scales to capture both initial and long‐term alterations in foraging ecology and habitat use. Fecal isotopes can provide a nearly instantaneous understanding of what resources an organism is utilizing as well as the variation between individuals foraging at the same time within and between landscapes (Blumenthal et al., 2012; Phillips & O'Connell, 2016). This timescale may be especially appropriate in rapidly shifting and regenerating mosaic landscapes, such as the areas where some of the last solenodon populations persist despite burning of forests for charcoal and sharecropping (Lloyd & León, 2019). Fecal isotopes can be an important component of the practitioner's toolbox for monitoring populations and detecting potential threats or declines as disturbances unfold. Fully realizing the potential of this tool in broader conservation contexts requires future controlled feeding studies in captivity to determine Δ_diet–feces_ for a wider range of taxa (e.g., as in Montanari, 2017), as these trophic enrichment factors are necessary for isotopic mixing models and delimiting species interactions. Fecal isotope analysis can be expanded as a viable monitoring strategy for solenodons moving forward across Hispaniola's diverse environments and land use systems. We recommend such an approach for the Caribbean's other last surviving mammals, such as the Hispaniolan hutia, which are in urgent need of basic natural history studies and enhanced monitoring efforts before they are no longer considered “Last Survivors” but, rather, recent casualties.

## CONFLICT OF INTEREST

The authors declare no conflict of interest associated with this study.

## AUTHOR CONTRIBUTIONS

**Alexis M. Mychajliw:** Conceptualization (lead); Data curation (lead); Formal analysis (lead); Funding acquisition (lead); Investigation (equal); Methodology (equal); Project administration (lead); Resources (lead); Visualization (lead); Writing – original draft (lead); Writing – review & editing (lead). **Juan N. Almonte:** Conceptualization (supporting); Investigation (supporting); Methodology (supporting); Resources (supporting). **Pedro A. Martinez:** Conceptualization (supporting); Investigation (supporting); Methodology (supporting); Resources (supporting). **Elizabeth A. Hadly:** Conceptualization (supporting); Funding acquisition (supporting); Methodology (supporting); Project administration (supporting); Resources (supporting); Supervision (equal); Writing – review & editing (supporting).

## Supporting information

Supplementary Material

## Data Availability

All relevant datasets are available within the Supporting Information.
