# Supplementary material for: Stable isotopes reveal seasonal dietary responses to agroforestry in a venomous mammal, the Hispaniolan solenodon (Solenodon paradoxus)
Source: Ecol Evol. 2022 Mar 24;12(3):e8761. doi: 10.1002/ece3.8761 (PMC8948124; doi:10.1002/ece3.8761)
Supplement: Supplementary file 1 — Supplementary Material [file ECE3-12-e8761-s001.docx]

**Supplemental Figures**


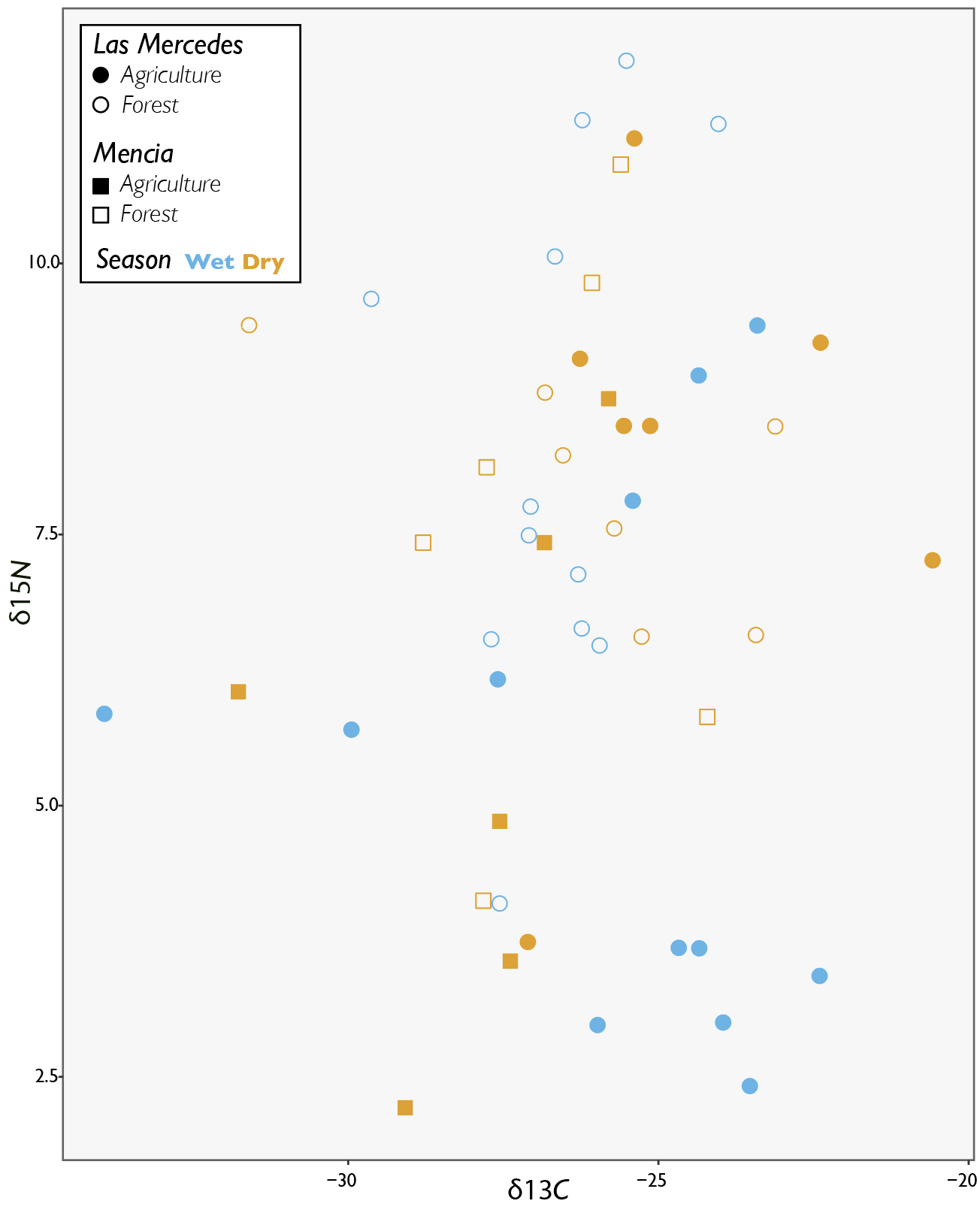


**Supplemental Fig 1.** δ^13^C and δ^15^N biplot of all fecal samples. Shapes represent sampling areas (Mencia, Las Mercedes), filled represent agriculture and open represent forests, and colors represent season of collection.


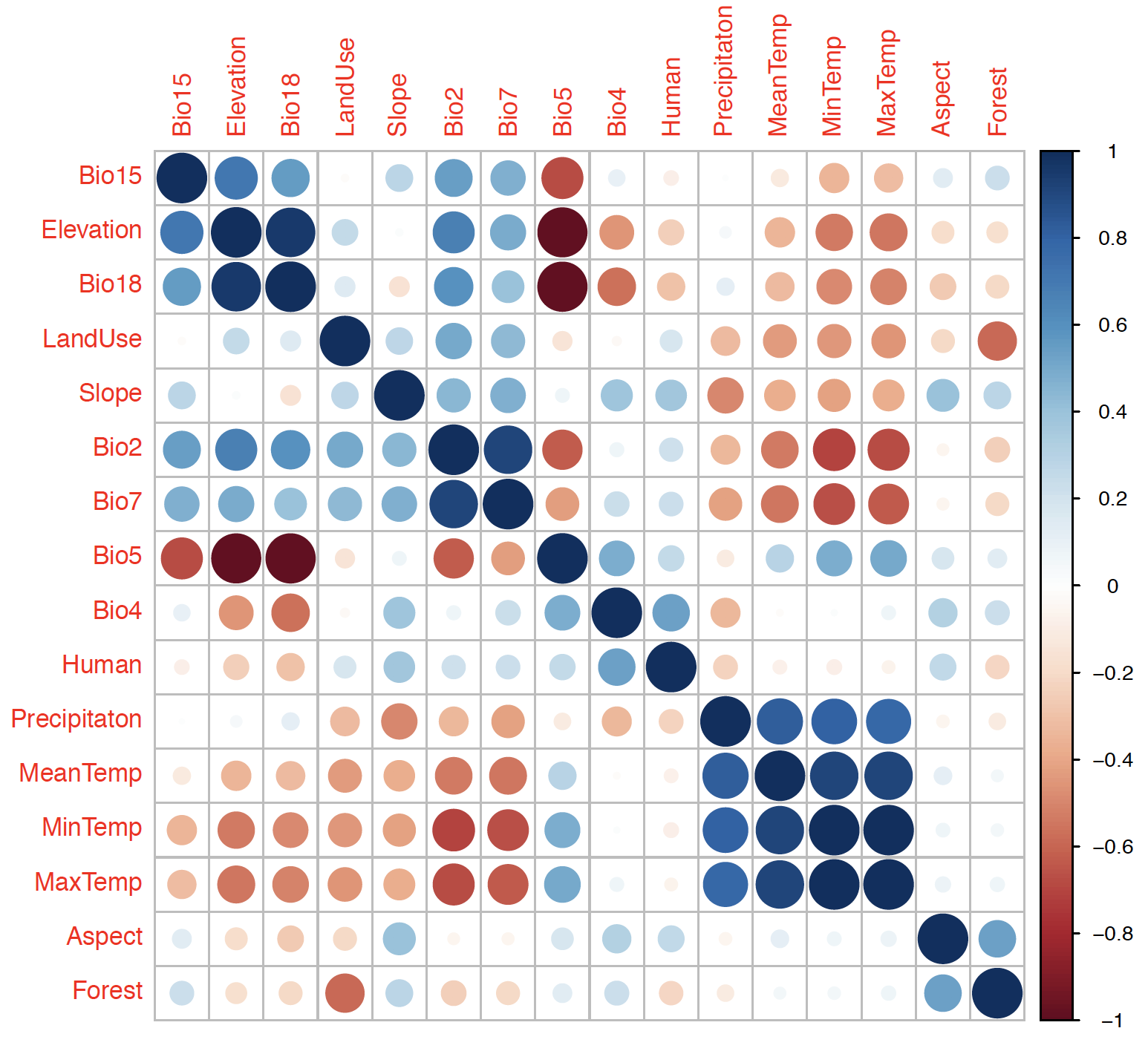


**Supplemental Fig 2.** Pearson correlations of environmental, anthropogenic, and climatic variables, ordered using the R package ‘hclust’.

**Supplemental Tables**

**Supplementary Table 1.** Summary of environmental conditions between dry and wet seasons by general sampling area, as given in mean ± standard deviation.

| **Site - Season** | **Elevation (m)** | **Slope** | **Min Temp (°C)** | **Max Temp**  **(°C)** | **Mean Temp (°C)** | **Precipitation**  **(mm)** |
| --- | --- | --- | --- | --- | --- | --- |
| Las Mercedes - Dry | 405. 64 ± 18.85 | 4.93 ± 5.12 | 16.27 ± 0.18 | 28. 63 ± 0.10 | 22.22 ± 0.88 | 27.71 ± 0.99 |
| Las Mercedes - Wet | 390. 38 ± 35.06 | 3.33 ± 2.37 | 19.75 ± 0.31 | 31. 28 ± 0.36 | 25.48 ± 0.36 | 79.33 ± 6.42 |
| Mencia  - Dry | 429 ± 185.47 | 15.92 ± 7.72 | 15.99 ± 1.66 | 28.58 ± 1.27 | 22.54 ± 1.09 | 27.5 ± 8.81 |

**Supplemental Table 2.** Raw (uncorrected) stable isotope values for the Hispaniolan solenodon (*Solenodon paradoxus*). *Samples MCZ 12313 and MCZ 3223 represent the Cuban almiqui (*Atopogale cubana*)*.* Sites are in the Dominican Republic unless otherwise noted.

| **Material** | **ID** | **Site** | **δ^15^N (‰)** | **δ^13^C (‰)** |
| --- | --- | --- | --- | --- |
| Hair | MCZ 34827 | La Vega | 5.16 | -23.73 |
| Hair | MCZ 34860 | La Vega | 4.37 | -22.90 |
| Hair | MCZ 34837 | La Vega | 5.92 | -20.95 |
| Hair | MCZ 34836 | La Vega | 6.72 | -20.95 |
| Hair | MCZ 34831 | La Vega | 4.80 | -22.99 |
| Hair | MCZ 34834 | La Vega | 6.24 | -20.95 |
| Hair | MCZ 34838 | La Vega | 5.78 | -16.11 |
| Hair | MCZ 34826 | La Vega | 5.99 | -22.54 |
| Hair | MCZ 34819 | La Vega | 5.72 | -23.53 |
| Hair | MCZ 34816 | La Vega | 5.79 | -22.65 |
| Hair | MCZ 12386 | La Vega | 5.63 | -24.72 |
| Hair | MCZ 12381 | La Vega | 5.82 | -20.74 |
| Hair | MCZ 12394 | La Vega | 5.69 | -21.67 |
| Hair | MCZ 12385 | La Vega | 4.86 | -22.97 |
| Hair | MCZ 12388 | La Vega | 7.63 | -20.15 |
| Hair | MCZ 12389 | La Vega | 7.11 | -23.92 |
| Hair | MCZ 34821 | La Vega | 6.12 | -23.18 |
| Hair | MCZ 34817 | La Vega | 5.63 | -22.62 |
| Hair | AMNH 35330 | Cordillera Central | 5.66 | -20.58 |
| Hair | USNM 217254 | La Vega | 4.79 | -21.62 |
| Hair | USNM 217256 | La Vega | 5.03 | -23.78 |
| Hair | USNM 217257 | La Vega | 5.24 | -21.65 |
| Hair | USNM 221030 | Samana | 9.11 | -24.55 |
| Hair | USNM 260142 | Santiago | 7.42 | -21.89 |
| Hair | MCZ 35313 | Sabana del Mar | 8.09 | -23.23 |
| Hair | MCZ 35312 | Sabana del Mar | 7.51 | -22.08 |
| Hair | UF 18820 | Haiti | 3.79 | -24.06 |
| Hair | MCZ 12313* | Cuba | 7.86 | -19.98 |
| Hair | MCZ 3223* | Cuba | 4.53 | -22.24 |
| Hair | AMNH 185012 | Zoo | 7.54 | -20.66 |
| Hair | USNM 364952 | Zoo | 6.53 | -18.36 |
| Hair | USNM 395819 | Zoo | 7.84 | -19.86 |
| Feces | MCigua1 | Las Mercedes | 9.27 | -22.39 |
| Feces | MCigua2A | Las Mercedes | 3.74 | -27.10 |
| Feces | MCigua3 | Las Mercedes | 8.50 | -25.56 |
| Feces | MCigua4 | Las Mercedes | 8.50 | -25.13 |
| Feces | MCigua5 | Las Mercedes | 9.12 | -26.26 |
| Feces | MCigua6 | Las Mercedes | 11.15 | -25.39 |
| Feces | MCigua7 | Las Mercedes | 7.26 | -20.58 |
| Feces | MElNu1 | Las Mercedes | 8.50 | -23.12 |
| Feces | MElNu1A | Las Mercedes | 7.56 | -25.71 |
| Feces | MElNu2 | Las Mercedes | 6.56 | -25.27 |
| Feces | MElNu3 | Las Mercedes | 8.81 | -26.83 |
| Feces | CdeLeon1 | Las Mercedes | 9.43 | -31.60 |
| Feces | CdeLeon3B | Las Mercedes | 8.23 | -26.54 |
| Feces | CdeLeon4 | Las Mercedes | 6.57 | -23.43 |
| Feces | SumCigua1 | Las Mercedes | 9.43 | -23.41 |
| Feces | SumCigua10 | Las Mercedes | 7.81 | -25.41 |
| Feces | SumCigua11 | Las Mercedes | 3.69 | -24.67 |
| Feces | SumCigua12 | Las Mercedes | 5.85 | -33.93 |
| Feces | SumCigua2 | Las Mercedes | 3.00 | -23.96 |
| Feces | SumCigua3 | Las Mercedes | 3.43 | -22.40 |
| Feces | SumCigua4 | Las Mercedes | 6.16 | -27.59 |
| Feces | SumCigua5 | Las Mercedes | 8.97 | -24.35 |
| Feces | SumCigua6 | Las Mercedes | 3.68 | -24.34 |
| Feces | SumCigua7 | Las Mercedes | 2.98 | -25.98 |
| Feces | SumCigua8 | Las Mercedes | 5.70 | -29.95 |
| Feces | SumCigua9 | Las Mercedes | 2.41 | -23.53 |
| Feces | SumElNu1 | Las Mercedes | 6.48 | -25.95 |
| Feces | SumElNu2 | Las Mercedes | 6.63 | -26.24 |
| Feces | SumElNu3 | Las Mercedes | 7.13 | -26.29 |
| Feces | SumElNu4A | Las Mercedes | 6.53 | -27.69 |
| Feces | SumElNu5 | Las Mercedes | 10.06 | -26.67 |
| Feces | SumLechosa1 | Las Mercedes | 11.87 | -25.52 |
| Feces | SumLechosa2 | Las Mercedes | 9.67 | -29.63 |
| Feces | SumLechosa3 | Las Mercedes | 11.32 | -26.23 |
| Feces | SumLechosa4 | Las Mercedes | 7.76 | -27.06 |
| Feces | SumLechosa5 | Las Mercedes | 4.10 | -27.56 |
| Feces | SumLechosa6 | Las Mercedes | 11.28 | -24.03 |
| Feces | SumLechosa7 | Las Mercedes | 7.49 | -27.09 |
| Feces | MSergio1 | Mencia | 4.86 | -27.56 |
| Feces | MSergio3 | Mencia | 8.75 | -25.80 |
| Feces | CdeBanano1 | Mencia | 6.05 | -31.77 |
| Feces | CdeBanano2 | Mencia | 3.57 | -27.39 |
| Feces | RioAguaNegra2 | Mencia | 2.21 | -29.08 |
| Feces | MSergio4 | Mencia | 7.42 | -26.84 |
| Feces | Manguito1 | Mencia | 10.91 | -25.61 |
| Feces | Manguito2 | Mencia | 5.82 | -24.21 |
| Feces | Manguito3A | Mencia | 9.82 | -26.07 |
| Feces | Manguito4 | Mencia | 8.12 | -27.77 |
| Feces | MMoncho1 | Mencia | 7.42 | -28.79 |
| Feces | RioAguaNegra1 | Mencia | 4.12 | -27.82 |

**Supplemental Table 3.** AIC values for each step in the stepwise regression with each dropped variable, as performed using the R package “MASS”.

| **Nitrogen: δ^15^N ~** | **AIC** |
| --- | --- |
| Slope + Aspect + Bio18 + Bio2 + Bio15 + Bio4 + MeanTempSeason +  Forest + LandUse + Human + dC | 92.99 |
| Slope + Aspect + Bio18 + Bio2 + Bio15 + Bio4 + MeanTempSeason +  Forest + LandUse + Human | 90.99 |
| Slope + Bio18 + Bio2 + Bio15 + Bio4 + MeanTempSeason + Forest +  LandUse + Human | 88.99 |
| Slope + Bio18 + Bio2 + Bio15 + Bio4 + MeanTempSeason + Forest +  Human | 87.1 |
| Slope + Bio18 + Bio2 + Bio15 + Bio4 + MeanTempSeason + Human | 85.64 |
| Slope + Bio18 + Bio2 + Bio4 + MeanTempSeason + Human | 84.36 |
| Slope + Bio2 + Bio4 + MeanTempSeason + Human | 82.56 |
| Bio2 + Bio4 + MeanTempSeason + Human | 81.9 |
| **Carbon: δ^13^C ~** | **AIC** |
| Slope + Aspect + Bio18 + Bio2 + Bio15 + Bio4 + MeanTempSeason +  Forest + LandUse + Human + dN | 99.31 |
| Slope + Aspect + Bio18 + Bio2 + Bio15 + Bio4 + MeanTempSeason +  Forest + LandUse + Human | 97.31 |
| Slope + Aspect + Bio18 + Bio15 + Bio4 + MeanTempSeason +  Forest + LandUse + Human | 95.36 |
| Slope + Bio18 + Bio15 + Bio4 + MeanTempSeason + Forest +  LandUse + Human | 93.7 |
| Slope + Bio18 + Bio4 + MeanTempSeason + Forest + LandUse +  Human | 92.62 |
| Bio18 + Bio4 + MeanTempSeason + Forest + LandUse + Human | 91.35 |
| Bio18 + MeanTempSeason + Forest + LandUse + Human | 90.54 |

**Supplemental Table 4.** Comparison of initial model (all variables included) with final model selected based on lowest AIC values, as generated using stepwise regression. *p<0.1; **p<0.05; ***p<0.01

|  | **δ^15^N** | | **δ^13^C** | |
| --- | --- | --- | --- | --- |
| **Variable** | *Initial* | *Final* | *Initial* | *Final* |
| Slope | 0.086 | - | -0.081 |  |
| Aspect | -0.0002 | - | 0.003 |  |
| Bio18 | 0.014 | - | -0.026 | -0.012 |
| Bio2 | -0.858 | -0.766*** | -0.109 |  |
| Bio15 | -0.645 | - | 0.682 |  |
| Bio4 | 0.084* | 0.071*** | -0.046 |  |
| MeanTempSeason | -0.283 | -0.420* | -0.683** | -0.463* |
| Forest | 0.004 | - | -0.018 | -0.021** |
| LandUse | -0.026 | - | -0.089 | -0.127 |
| Human | -0.001* | -0.0005** | -0.0003 | -0.001** |
| dC | -0.0002 | - | - | - |
| dN | - | - | -0.0003 | - |
| Constant | 45.66 | 24.22 | 31.952 | -7.446 |
| Observations | 50 | 50 | 50 | 50 |
| R2 | 0.383 | 0.346 | 0.246 | 0.195 |
| Adjusted R2 | 0.204 | 0.288 | 0.027 | 0.104 |
| Residual Std. Error | 2.287 (df = 38) | 2.163 (df = 45) | 2.436 (df = 38) | 2.338 (df = 44) |
| F Statistic | 2.143** (df = 11; 38) | 5.947*** (df = 4; 45) | 1.126 (df = 11; 38) | 2.138* (df = 5; 44) |
